# Structural disconnection and functional reorganization in Fabry disease: a multimodal MRI study

**DOI:** 10.1093/braincomms/fcac187

**Published:** 2022-07-22

**Authors:** Ilaria Gabusi, Giuseppe Pontillo, Maria Petracca, Matteo Battocchio, Sara Bosticardo, Teresa Costabile, Alessandro Daducci, Chiara Pane, Eleonora Riccio, Antonio Pisani, Arturo Brunetti, Simona Schiavi, Sirio Cocozza

**Affiliations:** Department of Computer Science, Diffusion Imaging and Connectivity Estimation (DICE) Lab, University of Verona, Verona 37134, Italy; Department of Advanced Biomedical Sciences, University “Federico II”, Naples 80131, Italy; Department of Advanced Biomedical Sciences, University “Federico II”, Naples 80131, Italy; Department of Electrical Engineering and Information Technology (DIETI), University “Federico II”, Naples 80125, Italy; Department of Human Neuroscience, Sapienza University of Rome, Rome 00189, Italy; Department of Computer Science, Diffusion Imaging and Connectivity Estimation (DICE) Lab, University of Verona, Verona 37134, Italy; Department of Computer Science, University of Sherbrooke, Sherbrooke, QC J1K 2R1, Canada; Department of Computer Science, Diffusion Imaging and Connectivity Estimation (DICE) Lab, University of Verona, Verona 37134, Italy; Department of Biomedical Engineering, Translational Imaging in Neurology (ThINk), University Hospital Basel and University of Basel, Basel 4001, Switzerland; Department of Clinical and Experimental Medicine, Multiple Sclerosis Centre, II Division of Neurology, ‘'Luigi Vanvitelli” University, Naples 80138, Italy; Department of Computer Science, Diffusion Imaging and Connectivity Estimation (DICE) Lab, University of Verona, Verona 37134, Italy; Department of Neurosciences and Reproductive and Odontostomatological Sciences, University “Federico II”, Naples 80131, Italy; Department of Public Health, Nephrology Unit, University “Federico II”, Naples 80131, Italy; Department of Neurosciences and Reproductive and Odontostomatological Sciences, University “Federico II”, Naples 80131, Italy; Department of Advanced Biomedical Sciences, University “Federico II”, Naples 80131, Italy; Department of Computer Science, Diffusion Imaging and Connectivity Estimation (DICE) Lab, University of Verona, Verona 37134, Italy; Department of Neuroscience, Rehabilitation, Ophthalmology, Genetics, Maternal and Child Health (DINOGMI), University of Genoa, Genoa 16132, Italy; Department of Advanced Biomedical Sciences, University “Federico II”, Naples 80131, Italy

**Keywords:** Fabry disease, magnetic resonance imaging, brain connectivity, multimodal study, microstructure informed tractography

## Abstract

Central nervous system involvement in Fabry disease, a rare systemic X-linked lysosomal storage disorder, is characterized by the presence of heterogeneous but consistent functional and microstructural changes. Nevertheless, knowledge about the degree and extension of macro-scale brain connectivity modifications is to date missing. In this work, we performed connectomic analyses of diffusion and resting-state functional MRI to investigate changes of both structural and functional brain organization in Fabry disease, as well as to explore the relationship between the two and their clinical correlates. In this retrospective cross-sectional study, 46 patients with Fabry disease (28F, 42.2 ± 13.2years) and 49 healthy controls (21F, 42.3 ± 16.3years) were included. All subjects underwent an MRI examination including anatomical, diffusion and resting-state functional sequences. Images were processed to obtain quantitative structural and functional connectomes, where the connections between regions of interest were weighted by the total intra-axonal signal contribution of the corresponding bundle and by the correlation between blood-oxygen level–dependent time series, respectively. We explored between-group differences in terms of both global network properties, expressed with graph measures and specific connected subnetworks, identified using a network-based statistics approach. As exploratory analyses, we also investigated the possible association between cognitive performance and structural and functional connectome modifications at both global and subnetwork level in a subgroup of patients (*n* = 11). Compared with healthy controls, patients with Fabry disease showed a significantly reduced global efficiency (*P* = 0.005) and mean strength (*P* < 0.001) in structural connectomes, together with an increased modularity (*P* = 0.005) in functional networks. As for the network-based statistics analysis, a subnetwork with decreased structural connectivity in patients with Fabry disease compared with healthy controls emerged, with eight nodes mainly located at the level of frontal or deep grey-matter areas. When probing the relation between altered global network metrics and neuropsychological tests, correlations emerged between the structural and functional disruption with results at verbal and working memory tests, respectively. Furthermore, structural disruption at subnetwork level was associated with worse executive functioning, with a significant moderation effect of functional changes suggesting a compensation mechanism. Taken together, these results further expand the current knowledge about brain involvement in Fabry disease, showing widespread structural disconnection and functional reorganization, primarily sustained by loss in axonal integrity and correlating with cognitive performance.

## Teaser

Gabusi *et al*. examined structural and functional changes of brain connectivity in Fabry disease using diffusion and resting-state functional MRI data. The connectome analysis of brain networks revealed that patients with Fabry disease show both a structural disconnection (due to mild but widespread axonal damage) and a functional reorganization, associated to cognitive performances.

## Graphical Abstract

**Graphical abstract:**
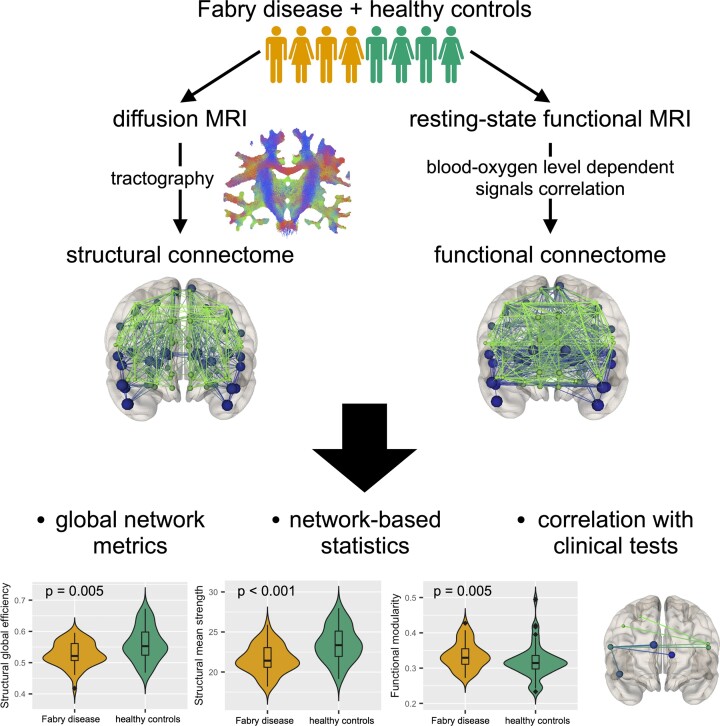

## Introduction

Fabry disease is a rare X-linked lysosomal storage disease characterized by a defective activity of the α-galactosidase A (α-GalA) enzyme, which leads to an intracellular accumulation of the glycosphingolipid globotriaosylceramide (Gb3) in different cells.^1^ Neurological manifestations have been long considered the consequence of endothelial dysfunction^2^ leading to neurovascular events.^3^ However, although the development of white-matter (WM) hyperintensities secondary to cerebral small vessel disease remains the most frequent finding in patients with Fabry disease,^4,5^ recent evidences have suggested the presence of a more profound and complex CNS involvement.^6^ Over the last decade, volumetric,^7,8^ functional^9,10^ and microstructural^11–13^ changes have been described in patients with Fabry disease. In particular, widespread WM microstructural damage has been reported in whole-brain analyses^9,13^ also in patients without significant WM lesion load^14^ and in extra-lesional WM,^13^ while microstructural WM changes associated to functional rearrangements have been documented for specific circuits.^10,12^ Such profound involvement of the WM compartment likely affects the overall brain organization, in terms of structural and functional interconnection between brain regions. Indeed, although limited to relatively few evidence, diffuse and significant functional changes affecting both the motor and the cognitive domains seem to be present in Fabry disease. In particular, a task-based study^15^ showed a recruitment of additional cortical areas during a simple motor task, with a resting-state functional MRI (RS-fMRI) study that confirmed the presence of this subclinical involvement of motor circuits.^10^ Furthermore, this approach has also been used to demonstrate the presence of changes occurring in the cognitive domain, with a reported increase of the functional connectivity between the main hubs of the default mode network (DMN).^9^

**Figure 1:**
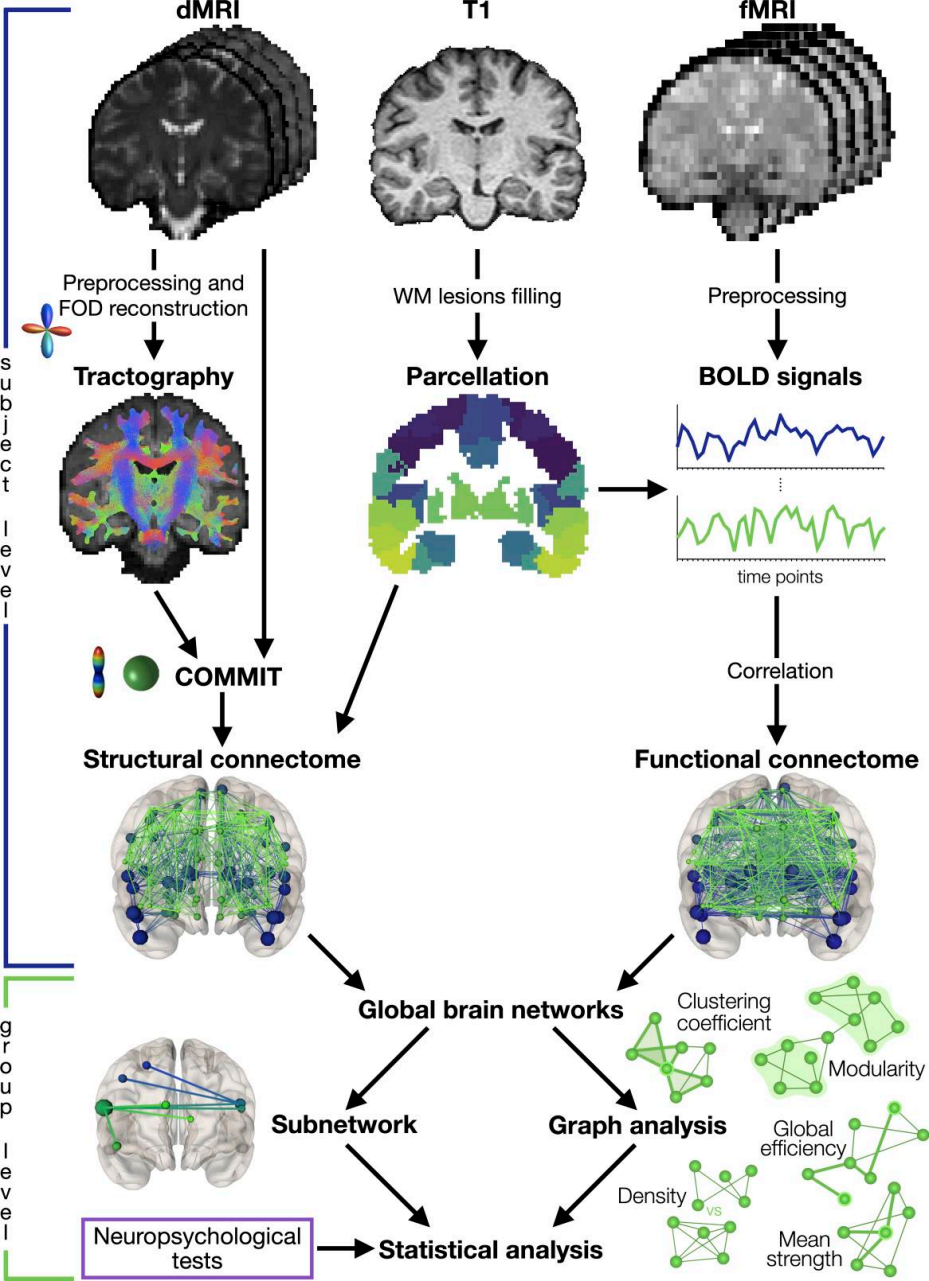
**Flow chart summarizing the main steps of this study.** Probabilistic tractography was performed and combined with the GM parcellation to identify the structural connectomes. Through COMMIT, they were quantified by exploiting the microstructure information included in the diffusion-weighted MRIs. In parallel, functional connectomes were obtained by analysing the correlation between BOLD signals of different regions of interest. From both types of networks (structural and functional), global graph metrics were extracted and evaluated as descriptors of brain connectivity. By means of NBS, a subnetwork associated with a significant between-group difference was extracted from the complete structural connectomes. Finally, possible associations between altered global network metrics and neuropsychological tests were investigated. dMRI, diffusion-weighted magnetic resonance imaging; FOD, fibre orientation distribution; COMMIT, Convex Optimization Modelling for Microstructure Informed Tractography; WM, white matter; fMRI, functional magnetic resonance imaging; BOLD, blood-oxygen level dependent; NBS, network-based statistics.

Connectomic analyses have been successfully applied to the description of other conditions characterized by focal and diffuse WM damage, such as multiple sclerosis^16^ and schizophrenia,^17^ and therefore appear as a promising tool for the characterization of brain architecture in Fabry disease. Indeed, the representation of the brain as a network of interconnected nodes offers the possibility to investigate the effects of pathology-specific damage on brain architecture both from a structural and functional perspective, according to the information selected to represent the links between segregated brain regions.^18,19^ To this aim, here we explored brain organization in Fabry disease, comparing synthetic measures derived from structural (diffusion based) and functional (resting-state fMRI based) connectomes and exploring the presence of disconnected subnetworks (Fig. 1). To test the clinical meaningfulness of these possible changes, we conducted an exploratory analysis of their impact on cognitive performance, while evaluating the interactions occurring between functional and structural changes. Our hypothesis was that the preservation of functional connectivity might attenuate the impact of possible structural network disruption on cognition.

## Materials and methods

### Participants

In this retrospective cross-sectional study, part of a larger monocentric framework on the involvement of CNS in Fabry disease (FD), patients with a genetic diagnosis were selected, along with age- and sex-comparable healthy controls (HCs). Only subjects with age ≥18 years were included in the study. To avoid the confounding effect of major cerebrovascular events, participants with a history of stroke or transient ischaemic attacks were not included in this study. Additional exclusion criteria included left-handedness and the presence of other relevant neurological, psychiatric or systemic conditions that could affect the CNS.

For all patients with Fabry disease, clinical variables of systemic organ involvement were obtained from medical records, and included the following: diabetes mellitus, hypertension, cardiac arrhythmia, left ventricular hypertrophy, renal failure (for estimated glomerular filtration rates <90 mL/min), proteinuria (for scores >150 mg/24 h), cephalalgia and acroparesthesia.

When available, results of a neuropsychological examination obtained within 1 week from the MRI scan were also retrieved and standardized for age and education. In particular, mini mental state evaluation (MMSE),^20^ Rey Auditory Verbal Learning Test (RAVLT),^21^ Corsi block-tapping test (CBTT),^22^ digit span (DGS) test,^22^ attentional matrices^23^ and Weigl colour form sorting test (WCFST)^22^ were included as measures of general cognition, immediate and delayed verbal memory, visual memory, working memory, attention and executive function, respectively.

The study was conducted in compliance with ethical standards and approved by the local ethics committee. Written informed consent was obtained from all subjects according to the Declaration of Helsinki.

### MRI data acquisition

All MRI examinations were performed on the same 3 T scanner (Magnetom Trio, Siemens Healthineers), equipped with an eight-channel head coil. The acquisition protocol included: a structural T_1_-weighted volume acquired using a 3D magnetization prepared rapid acquisition gradient echo sequence (repetition time [TR] = 1900 ms; echo time [TE] = 3.4 ms; inversion time [TI] = 900 ms; flip angle 9°; voxel size 1 × 1×1 mm^3^; 160 axial slices), used as anatomical reference; a 3D fluid attenuated inversion recovery (FLAIR) sequence for the assessment of eventual white-matter lesions (WMLs; TR = 6000 ms; TE = 396 ms; TI = 2200 ms; flip angle = 120°; voxel size = 1 × 1 × 1 mm^3^; 160 sagittal slices); diffusion tensor images acquired using a spin echo-planar imaging (EPI) sequence (TR = 7400 ms; TE = 88 ms; flip angle = 90°; voxel size = 2.2 × 2.2 × 2.2 mm^3^ with 64 directions at *b* = 1000 s/mm^2^ in addition to 9 *b* = 0 s/mm^2^; 60 axial slices; GRAPPA acceleration factor = 2), for the structural connectivity analysis; T_2_*-weighted volumes acquired using a gradient echo EPI sequence (TR = 2500 ms; TE = 50 ms; voxel size = 3 × 3 × 4 mm^3^; gap = 1 mm; 200 time points; 30 axial slices; GRAPPA acceleration factor = 1), for the functional connectivity analysis.

### MRI data processing

#### Anatomical images

The presence of WML in patients with Fabry disease was investigated on FLAIR images by an experienced neuroradiologist with more than 10 years of experience in the field of neuroimaging (S.C.), and graded according to a modified Fazekas score,^24^ in line with previous studies.^9,25^ Furthermore, to correct for the potential impact of WML on anatomical image processing, lesions were segmented using a semi-automated approach (Jim 7; http://www.xinapse.com/home.php) and the resulting masks employed for correcting lesion intensities in T_1_-weighted volumes according to the filling procedure implemented in FSLv5.0.10 (FMRIB Software Library; http://www.fmrib.ox.ac.uk/fsl). To obtain the grey-matter (GM) regions of interest (ROI) that serve as nodes to build the connectomes, a non-linear registration was estimated using the Computational Anatomy Toolbox (CAT12.6, http://www.neuro.uni-jena.de/cat) for statistical parametric mapping (SPM) and used to transform the automated anatomical labelling atlas,^26^ available in the MNI152 space, to each subject’s T_1_-weighted volume. Then, because of the low spatial resolution of EPIs, cerebellar ROIs were redefined by creating a unique region for the vermis and by merging the homologous regions in the two cerebellar hemispheres. At the end of this process, the total number of ROIs used in the subsequent analyses was 100 compared with the original 116.

To perform whole-brain anatomically constrained tractography^27^ using MRtrix3,^28^ both the five tissue type (5TT) image and the mask corresponding to the interface between WM and GM (gmwmi) were segmented from the T_1_-weighted image accounting for lesions. Then, T_1_-weighted, 5TT and gmwmi images, along with the parcellation, were registered in the subject-specific diffusion-weighted image (DWI) space using FMRIB’s Linear Image Registration Tool, FLIRT (FSL, https://fsl.fmrib.ox.ac.uk), with boundary-based cost function^29^ and nearest neighbour as interpolator.

#### Diffusion MRI

The quality of the DWI sequence was improved by removing the noise^30^ and by correcting for movement artefacts and distortions due to eddy currents.^31^ The fibre orientation distribution (FOD) functions were computed using constrained spherical deconvolution.^32^ Then, 3 million streamlines were generated using the probabilistic algorithm iFOD2^33^ and filtered to keep only those connecting GM ROIs. The resulting tractograms were processed using the Convex Optimization Modelling for Microstructure Informed Tractography (COMMIT).^34,35^ Briefly, by employing the ball and stick model,^36^ COMMIT assumes that the diffusion signal is due to water molecules present in two specific compartments: the restricted region inside axons and the isotropic areas (CSF contamination, GM partial volume and lesions). The signal is therefore decomposed into the intra-axonal (modelled by stick with axial diffusivity equal to 1.7 × 10^−3^ mm^2^/s and null perpendicular diffusivity) and the isotropic (represented by ball with two different diffusivities of 1.7 × 10^−3^ mm^2^/s and 3.0 × 10^−3^ mm^2^/s^37^) contributions. The properties of microstructure and, consequently, the restricted signal contribution, are assumed to be constant along the entire fibre trajectory, whereas the extra-axonal contribution is specific for each voxel.

The COMMIT’s weights, reflecting the intra-axonal signal contribution of each tract to the measured diffusion signal, can be used as a proxy for the connection strength in the structural connectomes of the subjects. With this approach, that already proved to be robust and reliable in a condition characterized by the presence of WM lesions,^16^ each connection was weighted by the total signal fraction associated to the corresponding bundle of streamlines. To each edge (*a*_*ij*_) was assigned the weighted average intra-axonal signal contribution of the corresponding bundle, obtained dividing the sum of COMMIT’s weights along the streamlines’ length by the average length of the bundle:

$$a_{ij}=\frac{\sum_{k=1}^{N_{ij}}\;x_{ij}^{k}l_{k}}{\frac{\sum_{k=1}^{N_{ij}}\;l_{k}}{N_{ij}}}$$

where *N*_*ij*_ is the number of streamlines in the bundle connecting the ROIs *i* and *j*, $x_{ij}^{k}$ is the weight given by COMMIT to the streamline *k* and *l*_*k*_ is its length. Finally, to remove possibly residual spurious connections, the structural connectomes were filtered using proportional thresholding: the connections present in less than half of the HCs were removed.^38^

#### Functional MRI

RS-fMRI data were processed using the FC toolbox (CONN, v.19.b, http://www.nitrc.org/projects/conn),^39^ which contains libraries for fMRI analysis based on the SPM software package (https://www.fil.ion.ucl.ac.uk/spm). Pre-processing steps included motion correction, slice-timing correction, outlier identification, indirect segmentation and normalization to the MNI152 space (consisting of functional/anatomical registration, segmentation and normalization using structural T_1_-weighted volumes and application of the estimated non-linear transformation to functional data) and resampling to 2 mm isotropic voxels.^40^ In order to minimize the residual non-neural variability of functional data, additional denoising steps were also applied, including linear regression of potential confounding effects (i.e. noise components from WM and CSF areas^41^ estimated subject–motion parameters, identified outlier scans as per the ‘scrubbing’ procedure^42^), and temporal band-pass filtering (0.008 Hz < *f* < 0.09 Hz). From pre-processed RS-fMRI data, symmetric ROI-to-ROI connectivity matrices were obtained, with edges defined as the Fisher-transformed bivariate correlation coefficient between the blood-oxygen level–dependent (BOLD) time series (after convolution with the canonical haemodynamic response function) extracted from each pair of atlas-defined ROIs. Finally, before entering graph analysis, the raw matrices were absolutized as inverse correlations may encode relevant information and most network metrics do not take into account negative values,^43,44^ and filtered using proportional thresholding (with a fixed network density of 0.20) to reduce the chance of spurious connections.^45^

### Graph measures

For each subject, five global network metrics were extracted from both structural and functional connectomes using the python version of the Brain Connectivity Toolbox (https://github.com/aestrivex/bctpy).^46^ These were the *density* (defined as the fraction of the actual edges to all possible connections in the network), the *mean strength* (corresponding to the average of all nodal strengths, where the nodal strength is the sum of the weights of all edges in which the node participates), the *global efficiency* (computed as the average inverse shortest path length, and inversely related to the characteristic path length), the *clustering coefficient* (defined as the average of each node’s fraction of triangles over the entirety of the connected triplets) and the *modularity* (corresponding to the number of edges falling within groups minus the expected number in an equivalent network with edges placed at random, computed through Newman’s spectral community detection algorithm^47^).

### Statistical analysis

#### Between-group differences in network metrics

Unless otherwise specified, statistical analyses were carried out using the Statistical Package for Social Science (SPSSv25.0, IBM Corp.) with a significance level *α* = 0.05, and the Benjamini–Hochberg procedure was adopted for controlling the false discovery rate.

Between-group differences were tested with either Student’s *t* (age), Pearson χ^2^ (sex) or age and sex (and mean motion for functional connectome-related metrics) adjusted robust analysis of covariance (ANCOVA; graph measures) tests.

#### Network-based statistics

To identify specific connected subnetworks associated with a significant between-group difference, a network-based statistics (NBS) approach was adopted.^48^ The NBS is a non-parametric method for performing statistical analysis on large networks, that deals with the multiple comparisons problem by clustering in the topological rather than the physical space.^48^ A brief description of the method is here provided: (i) the hypothesis of interest is tested at every connection in the network using the general linear model (mass univariate testing); (ii) connections are filtered according to a test statistic threshold (i.e. only connections with a test statistic exceeding this primary threshold are admitted to subsequent steps); (iii) connected graph components are identified among supra-threshold connections; (iv) a family-wise error rate (FWER)-corrected *P*-value is computed for each component based on its size (measured in terms of component extent − the total number of connections or component intensity − the sum of test statistic values across all its connections) using permutation testing.^48^

The analysis was performed using NBS Connectome (v1.2; https://sites.google.com/site/bctnet/network-based-statistic-toolbox). Between-group differences (FD < HC and FD > HC contrasts) were tested on both structural and functional connectivity matrices for a wide range of primary thresholds (from *t* = 2.0 to *t* = 4.0)^49^ via ANCOVA analyses, with age, sex and mean motion (for functional connectivity matrices only) as nuisance variables. Five thousand permutations were used, with intensity as the measure of network size and a statistical significance threshold set at *P* < 0.05 (FWER corrected).

#### Correlation with neuropsychological tests

As exploratory analyses, we investigated the possible association between cognitive performance and structural and functional connectome modifications at both global and subnetwork level.

Global network metrics associated with a significant between-group difference were adjusted for the effect of age and gender (and mean motion for RS-fMRI-derived metrics), as measured in the HC group, and the relationship between the resulting *z*-scores and neuropsychological tests was assessed via robust correlation analyses, correcting for age and gender and using a bootstrap approach with 1000 replications.

As for the subnetworks identified at the NBS analysis, we adapted a previously described approach.^50^ Briefly, a measure of mean structural connectivity disruption was obtained by referencing each subnetwork edge’s weight to HC values (adjusting for age and gender) and averaging the resulting *z*-scores. Similarly, an index of mean within-subnetwork functional connectivity deviation was obtained by averaging the *z*-scores of each edge’s absolute weights referenced to HC values (adjusting for age, gender and mean motion), so that positive values reflect increased functional connectivity with respect to HC and vice versa.^50^ Firstly, the correlation between neuropsychological test scores and structural network disruption was tested. When significant correlations emerged, a moderated multiple linear regression analysis predicting the cognitive score of interest was conducted, with structural network disruption, functional network deviation and their interaction as independent variables and age and gender as additional covariates.

Specifically, the interaction term was intended to test the hypothesis that functional connectivity preservation/increase could attenuate the impact of structural network disruption on cognition. Given their exploratory nature, these analyses were not adjusted for multiple comparisons.

### Data availability

The data evaluated in this study are available upon reasonable request to the corresponding author and following unanimous approval from all the co-authors.

## Results

### Participants

Forty-six patients (42.2 ± 13.2 years, M/F = 18/28) with genetic confirmed diagnosis of Fabry disease (95.7% classic, 4.3% non-classic) and 49 age- and sex-comparable HC (42.3 ± 16.3 years; M/F = 28/21) were included in this study. A complete list of the demographic, clinical and neuroradiological characteristics of the studied population is available in Table 1.

**Table 1:** Demographic and clinical characteristics of the studied population

	FD (*n* = 46)	HC (*n* = 49)	*P*-value
Age (year)^a^	42.2 ± 13.2	42.3 ± 16.3	0.98
Female sex	28 (60.9)	21 (42.9)	0.08
ERT	35 (76.1)	−	−
Cephalalgia	6 (13.0)	−	−
Acroparesthesia	7 (15.2)	−	−
Hypertension	12 (26.1)	−	−
Diabetes	1 (2.2)	−	−
Arrhythmia	3 (6.5)	−	−
Left ventricular hypertrophy	22 (47.8)	−	−
Renal failure	11 (23.9)	−	−
Proteinuria	17 (37.0)	−	−
White-matter lesion load^b^	1.52 ± 1.44 (0–6.72)	−	−
Fazekas score^c^	0 (0–2)	−	−
MMSE^a,d^	27.3 ± 2.8	−	−
RAVLT-immediate^a,d^	42.4 ± 7.0	−	−
RAVLT-delayed^a,d^	7.7 ± 2.8	−	−
CBTT^a,d^	4.7 ± 6.2	−	−
DGS test^a,d^	5.3 ± 0.7	−	−
AM^a,d^	41.9 ± 14.2	−	−
WCFST^a,d^	5.7 ± 2.8	−	−

Unless otherwise indicated, data are the number of subjects, with percentages in parentheses. Between-group differences were tested with either Student’s *t* (age) or Pearson χ^2^ (sex) tests.

FD, Fabry disease; HC, healthy controls; −, not applicable; ERT, enzyme replacement therapy; MMSE, mini mental state evaluation; RAVLT, Rey Auditory Verbal Learning Test; CBTT, Corsi block-tapping test; DGS, digit span test; AM, attentional matrices; WCFST, Weigl colour form sorting test.

^a^ Data are expressed in millilitres as mean ± standard deviation, with ranges in parentheses.

^b^ Data are expressed as mean ± standard deviation.

^c^ Data are median, with ranges in parentheses.

^d^ Neuropsychological examination was available only for 11 patients.

### Between-group differences in network metrics

**Figure 2:**
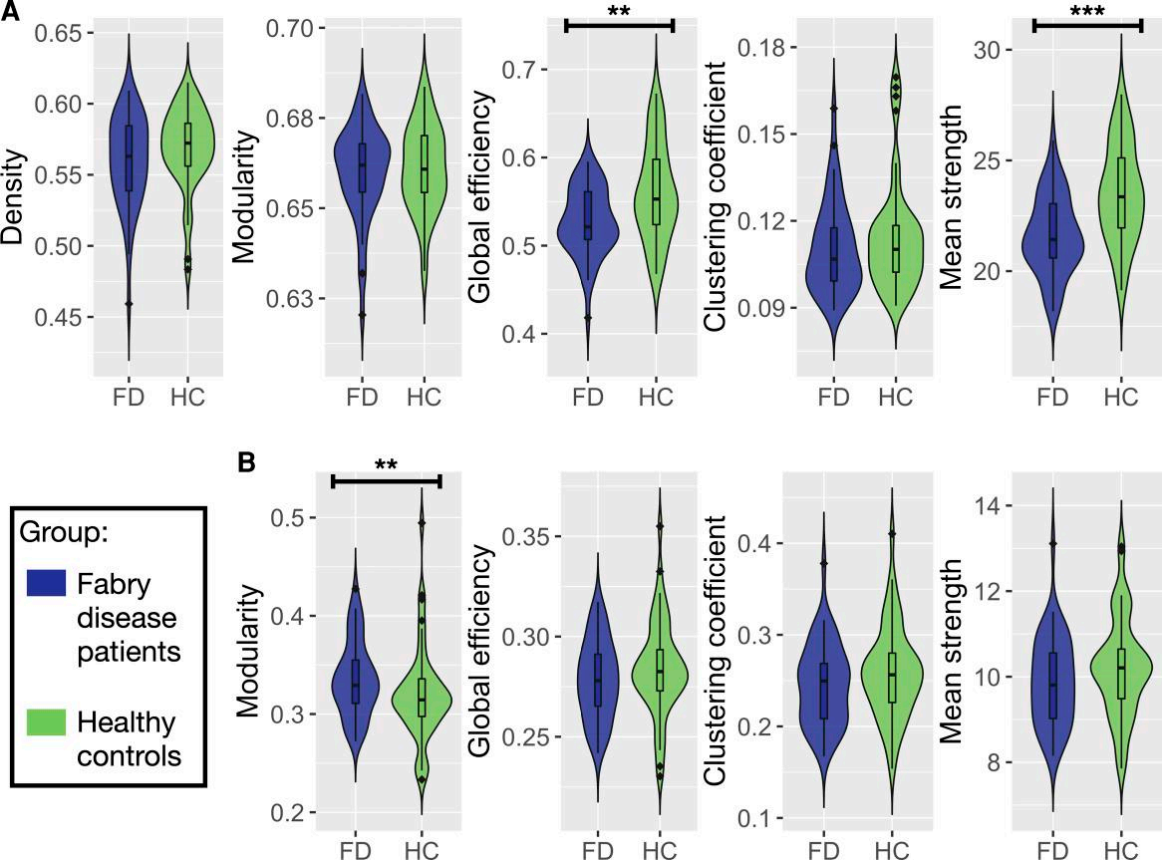
**Violin plots of global network metrics extracted for the structural and functional connectomes.** (**A**) Violin plots showing the differences in *structural* global network metrics (density, modularity, global efficiency, clustering coefficient and mean strength) between Fabry disease patients (FD, on the left) and healthy controls (HC, on the right). (**B**) Violin plots representing the distribution of the *functional* global network in the two groups of participants (FD and HC). Density is not reported because it was used to threshold the functional connectomes. Between-group differences between graph measures were tested with age and sex (and mean motion for functional connectome-related metrics) adjusted robust ANCOVA tests. Significance codes: ***0 ≤ *P* ≤ 0.001, **0.001 < *P* ≤ 0.01, *0.01 < *P* ≤ 0.05.

Results of the between-group comparisons in terms of graph measures for both the structural and functional networks are reported in Fig. 2 and Supplementary Table 1. Patients with Fabry disease showed reduced global efficiency (*P* = 0.005) and mean strength (*P* < 0.001) of the structural connectome compared with HC.

When looking at functional connectivity-related graph measures, patients with Fabry disease exhibited an increased modularity compared with HC (*P* = 0.005).

No significant between-group differences emerged for the remaining graph measures.

### Network-based statistics

**Figure 3:**
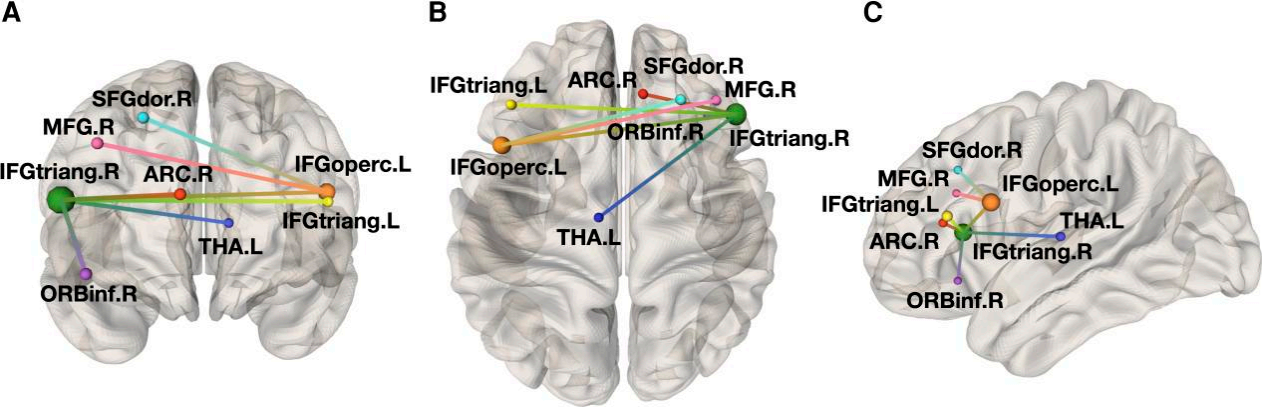
**Network-based statistics results.** Image shows coronal (**A**), axial (**B**) and sagittal (**C**) views of the subnetwork with decreased structural connectivity in Fabry disease patients (*N* = 46) compared with HCs (*N* = 49), emerging from the network-based statistics analysis with a primary threshold of *t* = 3.0. Its eight nodes, whose size reflects the number of their connections in the subnetwork (i.e. node’s degree), are: right superior frontal gyrus (SFGdor.R), right middle frontal gyrus (MFG.R), right inferior frontal gyrus—triangular part (IFGtriang.R), right inferior frontal gyrus—orbital part (ORBinf.R), right anterior cingulate and paracingulate gyri (ACG.R), left thalamus (THA.L), right inferior frontal gyrus—opercular part (IFGoperc.L) and left inferior frontal gyrus—triangular part (IFGtriang.L).

For the FD < HC contrast, the NBS analysis of structural connectomes revealed subnetworks associated with a significant between-group difference across a wide range of statistical thresholds (Table 2). No significant between-group differences emerged for the FD > HC contrast, or for the analysis of functional connectomes. The subnetwork identified at the median primary threshold of *t* = 3.0, offering a balanced representation of both topologically focal and distributed effects, and was considered for further analyses. This subnetwork, presented in Fig. 3, proved to be composed by eight nodes, involving mainly frontal areas such as the bilateral inferior frontal gyri, the right superior and middle frontal gyri and the cingulate and paracingulate cortices, as well as deep GM structures such as the left thalamus. A complete list of these nodes is available in Supplementary Table 2.

**Table 2:** Network-based statistics results at different primary thresholds for the FD < HC contrast

Primary threshold (*t*-value)	Nodes	Edges	*P*-value (FWER corrected)
2.0	94	200	0.009**
2.1	85	156	0.012*
2.2	78	127	0.013*
2.3	70	98	0.014*
2.4	55	71	0.016*
2.5	26	28	0.044*
2.6	–	–	ns
2.7	8	9	0.037*
2.8	8	7	0.023*
2.9	8	7	0.014*
3.0	8	7	0.009**
3.1	7	6	0.007**
3.2	6	5	0.006**
3.3	5	4	0.007**
3.4	4	3	0.006**
3.5	3	2	0.006**
3.6	2	1	0.005**
3.7	2	1	0.004**
3.8	2	1	0.004**
3.9	2	1	0.003**
4.0	2	1	0.003**

Significance codes: ***0 ≤ *P* ≤ 0.001, **0.001 < *P* ≤ 0.01, *0.01 < *P* ≤ 0.05.

ns, not significant.

### Correlation with neuropsychological tests

Results of the neuropsychological evaluation, available only for a subset of participants (*n* = 11), are reported in Table 1.

When investigating possible associations between altered global network metrics and neuropsychological tests, significant correlations emerged between the structural connectome mean strength and results obtained at the RAVLT-immediate score (*r* = 0.721, 95% confidence interval [CI]: 0.125, 0.949; *P* = 0.03), and between the modularity of functional connectomes and DGS test scores (*r* = −0.769, 95% CI: −0.979, −0.335; *P* = 0.02).

**Figure 4:**
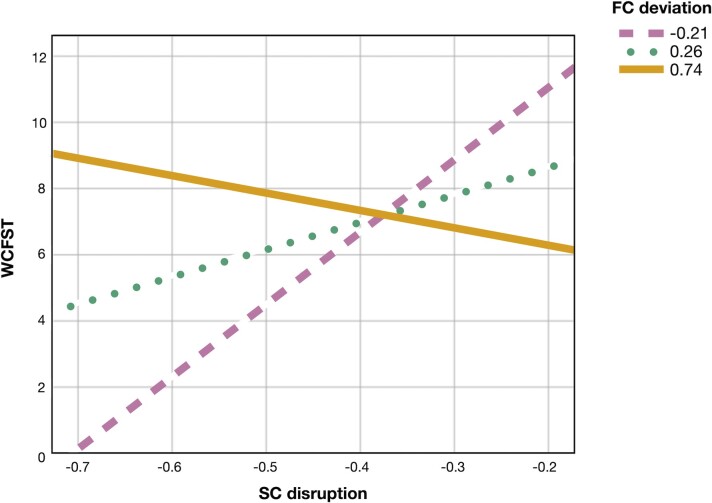
**Executive functioning and the structure−function interaction.** Plot shows the linear relationship between WCFST scores and structural connectivity disruption within the NBS-derived subnetwork at different levels of functional connectivity deviation (−0.21, 0.26 and 0.74, corresponding to −1 SD, mean and +1 SD of its distribution). Preserved functional connectivity tends to attenuate the impact of WM network disruption on executive functioning. The multiple linear regression model predicting WCFST scores was significant (*R*^2^ = 0.850, *P* = 0.04), with the following terms: constant (*β* = 16.364, 95% CI: 10.200, 22.528; SE = 2.395; *P* = 0.001); structural connectivity disruption (*β* = 15.809, 95% CI: 8.257, 23.360; SE = 2.934; *P* = 0.003); functional connectivity deviation (*β* = −10.793, 95% CI: −18.224, −3.361; SE = 2.887; *P* = 0.01); structural connectivity disruption*functional connectivity deviation (*β* = −28.636, 95% CI: −49.687, −7.584; SE = 8.178; *P* = 0.02); age (*β* = −0.070, 95% CI: −0.176, 0.037; SE = 0.041; *P* = 0.15); sex (*β* = −0.681, 95% CI: −4.099, 2.736; SE = 1.328; *P* = 0.63).

As for the subnetwork identified at the NBS analysis, the mean structural connectivity disruption across patients with Fabry disease was −0.62 ± 0.36 (versus 0.00 ± 0.62 in HCs; Cohen’s *d* = −1.22, *t* = 6.03, *P* < 0.001), with a mean functional connectivity deviation from HC of 0.14 ± 0.55 (versus 0.00 ± 0.42 in HCs; Cohen’s *d* = 0.29, *t* = 1.43, *P* = 0.16). Structural network disruption correlated with scores at the WCFST (*r* = 0.706, 95% CI: −0.102, 0.960; *P* = 0.03), the CBTT (*r* = 0.672, 95% CI: 0.037, 0.985; *P* = 0.05) and the RAVLT-delayed (*r* = 0.795, 95% CI: 0.115, 0.967; *P* = 0.01). In the regression model predicting WCFST scores (*R*^2^ = 0.850, *P* = 0.04; Fig. 4), statistically significant effects were observed for structural connectivity disruption (*β* = 15.809, 95% CI: 8.257, 23.360; SE = 2.934; *P* = 0.003), functional connectivity deviation (*β* = −10.793, 95% CI: −18.224, −3.361; SE = 2.887; *P* = 0.01) and the interaction between these variables (*β* = −28.636, 95% CI: −49.687, −7.584; SE = 8.178; *P* = 0.02). No significant moderation effects were observed for models predicting CBTT and RAVLT-delayed scores.

## Discussion

In this study, we compared functional and structural metrics in subjects with Fabry disease and HCs, showing the presence of widespread structural disconnection and functional reorganization in patients affected by this rare lysosomal storage disorder. In particular, we found a reduced mean strength and global efficiency within the structural connectome, and an increased modularity within the functional one. In addition to these global findings, we found the presence of a frontal subnetwork showing a severe structural disruption, with relatively preserved functional connectivity, showing some correlation with neuropsychological performance in a subset of patients. Overall, these findings support the presence of structural and functional disruption of the brain architecture in Fabry disease, sustained by axonal damage and associated to cognitive performance. Additionally, the analysis of the frontal subnetwork, more severely affected by the microstructural damage, offers insight on the compensatory role of functional connectivity reorganization on cognitive performance.

In particular, whole-brain connectome analyses revealed that, on average, patients with Fabry disease show an important decrease in terms of mean strength compared with healthy subjects, reflecting a reduction of the connection weights within Fabry disease connectomes. Since the connectome edges’ weight corresponds to the total signal fraction associated with the WM bundle, such decrease indicates a loss in axonal integrity. This interpretation is in agreement with preliminary data from neuropathological^51^ and animal study reports,^52^ documenting the presence of axonal damage in Fabry disease, either primary, related to glycolipid accumulation within neurons,^53^ or secondary to vasculopathy.^54^ The reduction in mean strength might also justify the observed decrease in global efficiency. Indeed, this metric measures the ability of the network to exchange information. Then, it follows that a damage to the brain axons determines a reduced efficiency of the connections and a partial loss of network integration. The generalized microstructural damage expressed by mean strength reduction was associated to worse performance at the verbal memory test. According to a recent model, verbal short-term memory relies on the integrity of a fronto-temporal sensory-motor circuit, whose core nodes are the superior temporal gyrus, the Sylvian-parietal-temporal region and the inferior frontal region,^55^ with the latter being a recurrent node within the structurally disconnected subnetwork we identified in Fabry disease.

From a functional perspective, such structural abnormalities are coupled with modifications in the network organization, resulting in the establishment of connections among nodes pertaining to the same functional module. Indeed, the modular organization of the human brain has been demonstrated by several functional neuroimaging studies, with each module displaying a group of densely interconnected nodes, which correspond to large-scale functional networks responsible for specialized tasks.^56–59^ In Fabry disease such modular organization is enhanced, and, of note, in our sample was associated with worse working memory. This is not surprising, as we assessed working memory via a digit symbol coding task, that requires the involvement of different neuronal circuits responsible for distinct but partly overlapping cognitive processes (lexical access speed, memory and information processing speed),^60^ and would thus likely be affected by the enhanced modular reorganization observed in Fabry disease. In agreement with this interpretation, previous studies have also highlighted a correlation between information processing speed, as measured via digit symbol coding, and WM integrity in Fabry disease,^13^ sporadic cerebral small vessel disease,^61–64^ and in cerebral autosomal dominant arteriopathy with subcortical infarcts and leukoencephalopathy (CADASIL).^64,65^

Within the whole structural connectome, we identified a disconnected subnetwork mainly pertaining to frontal areas. Although in Fabry disease, a prominent involvement of posterior brain areas has been previously reported,^13,66,67^ this refers to the localization of focal WML, hyperperfusion or metabolic disturbance, while microstructural damage is usually described as widespread and non-specifically localized within periventricular and deep WM,^11,13^ with relative sparing of the temporal and occipital lobes^9^ in whole-brain analysis, or localized to circuits involving frontal regions, when these are specifically investigated.^10,12^ Our approach, aimed to characterize the impact of WM damage on the interconnection between GM regions, offered results that are in line with the latter findings.^12^ We identified, within the globally affected connectome, a set of interconnected nodes characterized by severe structural disruption, associated with a relative preservation of functional connectivity. While the subnetwork structural disruption negatively affected verbal and visual memory, its effect on executive functions was attenuated by the preservation of functional connectivity, suggesting a possible compensatory mechanism. Indeed, while preservation in connectivity within a segregated circuit including frontal and deep GM areas might be enough to sustain the executive performance, it might not be enough to compensate for tasks requiring long-distance communication among secluded GM regions. This applies not only to verbal memory, which, as discussed above, relies on the connections between frontal and temporal areas, but also to visual memory, that, in Fabry disease, is directly affected by the functional connectivity between inferior frontal gyrus and precuneus.^9^

Our work is not without limitations. First, as mentioned in the Methods and Results sections, the correlation analysis with the neuropsychological tests was performed only in a subgroup of patients and should be therefore considered an exploratory investigation (*n* = 11). Further investigations involving a larger number of subjects with MRI and neuropsychological data are warranted to confirm these results. Furthermore, we did not perform neuropsychological evaluations in our cohort of HCs, and therefore, we were not able to define the presence of cognitive impairment in the patient cohort. However, the presence of cognitive deficits in Fabry disease has been already reported by several groups,^13,68,69^ and in this work, our aim was to test associations between the disruption of brain organization and clinical performance, rather than analyse the structural and functional abnormalities underlying specific cognitive deficits. Second, we did not explore the role of enzyme replacement therapy (ERT) on the observed modifications. Previous data, however, do not suggest a direct effect of ERT on focal^5,70,71^ nor microstructural WM damage.^13^ Third, we excluded subjects with a history of stroke or transient ischaemic attacks and thus our findings might not be translatable to this category of patients. Finally, due to the small sample available for each condition, we could not investigate the role of specific Fabry disease systemic manifestations, such as diabetes or hypertension, in driving small vessel disease pathology and, in turn, WM disruption.

Notwithstanding these limitations, we demonstrated the presence of structural and functional reorganization of brain architecture in Fabry disease, sustained by loss in axonal integrity and directly related to clinical performance. Larger, multicentre studies will be needed to clarify the presence of primary versus secondary neuronal involvement in Fabry disease, with possible consequences on the therapeutic management of this condition.

## Supplementary Material

fcac187_Supplementary_Data

## Data Availability

The data underlying this article will be shared on reasonable request to the corresponding author.

## Abbreviations

AM =: attentional matrices
ANCOVA =: analysis of covariance
BOLD =: blood-oxygen level dependent
CBTT =: Corsi block-tapping test
CI =: confidence interval
COMMIT =: Convex Optimization Modelling for Microstructure Informed Tractography
DGS =: digit span test
DWI =: diffusion-weighted image
EPI =: echo-planar imaging
ERT =: enzyme replacement therapy
FLAIR =: fluid attenuated inversion recovery
FOD =: fibre orientation distribution
FWER =: family-wise error rate
GM =: grey matter
gmwmi =: grey matter–white matter interface
HCs =: healthy controls
MMSE =: mini mental state evaluation
NBS =: network-based statistics
RAVLT =: Rey Auditory Verbal Learning Test
ROI =: region of interest
RS-fMRI =: resting-state functional MRI
SPM =: statistical parametric mapping
TE =: echo time
TI =: inversion time
TR =: repetition time
WCFST =: Weigl colour form sorting test
WM =: white matter
WML =: white-matter lesions
5TT =: five tissue type
