# Supplementary material for: Structural disconnection and functional reorganization in Fabry disease: a multimodal MRI study
Source: Brain Commun. 2022 Jul 22;4(4):fcac187. doi: 10.1093/braincomms/fcac187 (PMC9327118; doi:10.1093/braincomms/fcac187)
Supplement: fcac187_Supplementary_Data [file fcac187_supplementary_data.docx]

**Supplementary material**

**Supplementary Table 1 Descriptive statistics and analysis of global metrics from structural and functional networks**

|  | **FD (n=46)** | **HC (n=49)** | **p-value (FD vs HC)** |
| --- | --- | --- | --- |
| **Structural connectome** |  |  |  |
| Density | 0.560±0.032 | 0.569±0.028 | 0.14 |
| Modularity | 0.660±0.013 | 0,661±0.012 | 0.81 |
| Global efficiency | 0.527±0.038 | 0.560±0.054 | 0.005 ** |
| Clustering coefficient | 0.111±0.016 | 0,114±0.019 | 0.92 |
| Mean strength | 21.610±1.76 | 23.503±2.198 | < 0.001 *** |
| **Functional connectome** |  |  |  |
| Modularity | 0.336±0.038 | 0,319±0.047 | 0.005 ** |
| Global efficiency | 0.278±0.020 | 0.284±0.023 | 0.93 |
| Clustering coefficient | 0.245±0.044 | 0,258±0.050 | 0.93 |
| Mean strength | 9.887±1.032 | 10.187±1.145 | 0.76 |

Data are expressed as mean±standard deviation. Between-group differences were tested with robust ANCOVA analyses accounting for age and gender (and mean motion for functional connectome) as confounding factors.

Significance codes: ‘***’ 0≤p≤0.001, ‘**’ 0.001<p≤0.01, ‘*’ 0.01<p≤0.05

Abbreviations: FD Fabry disease, HC healthy controls

**Supplementary Table 2 Connections identified at network-based statistics analysis for a primary threshold of t=3.0, with anatomical labelling according to the AAL atlas.**

| **Node A** | **Node B** |
| --- | --- |
| Right Superior Frontal Gyrus | Left Inferior Frontal Gyrus (opercular part) |
| Right Middle Frontal Gyrus | Left Inferior Frontal Gyrus (opercular part) |
| Left Inferior Frontal Gyrus (opercular part) | Right Inferior Frontal Gyrus (triangular part) |
| Left Inferior Frontal Gyrus (triangular part) | Right Inferior Frontal Gyrus (triangular part) |
| Right Inferior Frontal Gyrus (triangular part) | Right Inferior Frontal Gyrus (orbital part) |
| Right Inferior Frontal Gyrus (triangular part) | Right Anterior Cingulate and Paracingulate Gyri |
| Right Inferior Frontal Gyrus (triangular part) | Left Thalamus |
